# Comparison of iron deficiency markers in patients with advanced heart failure

**DOI:** 10.1007/s00392-025-02775-5

**Published:** 2025-11-17

**Authors:** Maria Bakosova, Jan Krejci, Julius Godava, Eva Ozabalova, Hana Poloczkova, Tomas Honek, Peter Hude, Jan Machal, Helena Bedanova, Petr Nemec

**Affiliations:** 1 First Department of Internal Medicine—Cardioangiology, St. Anne’s University Hospital, Brno, Czech Republic; 2 Faculty of Medicine, Masaryk University, Brno, Czech Republic; 3 International Clinical Research Center, St Anne’s University Hospital, Brno, Czech Republic; 4 Center for Cardiovascular and Transplant Surgery, Brno, Czech Republic

**Keywords:** Iron deficiency, Heart failure, Soluble transferrin receptor, Ferritin, Transferrin saturation

## Abstract

**Introduction:**

Iron deficiency (ID) is a common comorbidity in patients with heart failure (HF). It is associated with reduced performance, frequent hospitalizations and low quality of life. There is an ongoing debate about the correct definition of ID in HF.

**Purpose:**

Refinement of the ID determination in patients with advanced HF. Evaluation of the association of iron metabolism parameters with other clinical characteristics.

**Patients and methods:**

One hundred-two patients with advanced HF were included, 48 in-patients, out-patients without LVAD and 25 of patients with LVAD implanted. In addition to conventional tests, we monitored iron metabolism parameters. The prevalence of ID was compared according to both the original and the updated ID definition. Correlations between individual iron markers and patients’ functional capacity were measured.

**Results:**

The prevalence of ID in patients with advanced HF was 65%. There was no statistically significant difference in individual iron parameters between the groups. All parameters except ferritin correlated with NT-proBNP in the in-patient group. Serum iron correlated most strongly with NT-proBNP and CRP across all groups. The sTfR parameter correlated the most with right heart catheterization parameters, serum iron and TSAT were the most specific parameters related to functional status.

**Conclusion:**

Patients with more advanced HF and severe limitation of functional capacity had worse iron parameters. Ferritin seems to be the less specific marker of ID, while detecting ID using serum iron and TSAT seems to be reliable. The correlation of ferritin with functional status or haemodynamics was the weakest of all the parameters studied.

**Graphical Abstract:**

**Figure:**
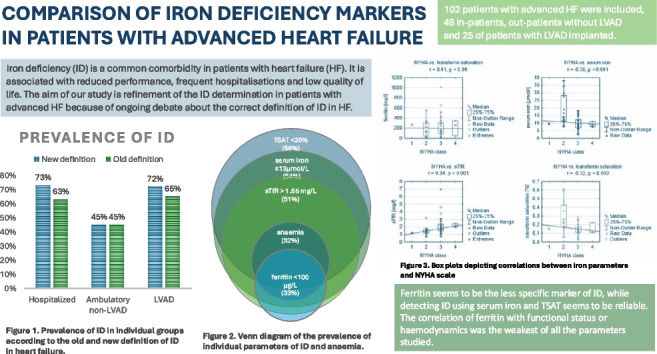

## Introduction

Iron deficiency (ID) is currently one of the most intensely discussed topics in the management of patients with heart failure (HF). It is one of the most common comorbidities in these patients and its presence significantly affects their quality of life and performance. ID is associated with more frequent hospitalizations for HF and, according to recent studies, with negative left ventricular remodelling [1, 2]. In out-patients with chronic HF, ID occurs in approximately 50% of patients, with a prevalence of up to 80% in acute HF, independent of the presence of anaemia [3]. ID is found across all HF phenotypes, with some studies suggesting a slightly higher prevalence in patients with heart failure with preserved ejection fraction (HFpEF) [4].

In patients with ID and HFrEF/HFmrEF, intravenous iron supplementation has been recommended since 2016 according to the HF Guidelines. This treatment leads to improvements in symptoms, quality of life and functional capacity in stable symptomatic HF patients with ID. In addition, it has been shown to reduce the risk of first hospitalization for worsening HF and to improve peak oxygen consumption on spiroergometry [5, 6]. Oral replacement is not effective in patients with HF as confirmed by the IRONOUT-HF study [7].

The AFFIRM-AHF trial provided data showing that administering of iron carboxymaltose (FCM) reduces the risk of repeated hospitalizations for HF in patients after an acute worsening of HF [8, 9]. These findings were reflected in the 2021 Guidelines for the Diagnosis and Management of Heart Failure (ESC Guidelines). The benefit and safety of intravenous iron supplementation in patients with HF was also demonstrated in the 2022 IRONMAN trial using a different molecule, iron desiromaltose [10].

Assessing the effect on mortality and performance, we can draw on the most recent evidence from the HEART-FID trial. This was a double-blind randomized trial that examined the number of deaths, hospitalizations for HF up to 12 months, and changes in the 6-min walk test (6MWT). The study confirmed the safety of the FCM administration and its effect on improving performance and reducing hospital admissions in patients with HF. It also brought an interesting news of the proper definition of a patient requiring the iron supplementation to the forefront [11].

Previously, ID in HF was defined as a ferritin value < 100 µg/l, or 100–299 µg/l in case transferrin saturation (TSAT) is < 20%. This definition reflects the criteria used by most studies testing iron administration, but not the pathophysiological contexts. It was found that serum ferritin levels individually are not prognostically significant in heart failure, whereas the TSAT < 20% or serum iron ≤ 13 μmol/l criteria express a worse prognosis [12–16]. Given the current state of knowledge, the definition of ID has been changed in the 2023 Focused Update of the 2021 ESC Guidelines as TSAT < 20% or ferritin < 100 µg/l.

## Purpose

The aim of our study was to find out the prevalence of ID in patients with advanced HF according to both the original and updated definitions of ID.

The next goal was to compare ID parameters between out-patients, patients hospitalized for worsening of HF and patients with left ventricle assist device (LVAD) implanted.

Furthermore, to compare ID parameters with other markers reflecting the severity of heart failure and the current state of the patient (laboratory methods, echocardiography, right heart catheterization, spiroergometry etc.), to evaluate which of the iron metabolism parameters is the most appropriate marker for definig the relationship between ID and the functional capacity of the patient.

### Study population

This observational, non‐interventional study was conducted at St Anne’s University Hospital Brno, 1st Internal Department – Cardioangiology. The study was approved by the institution´s Ethics committee. Data collection took place from March 2022 to January 2024.

We included 102 consecutive patients with advanced HF, 48 of whom were hospitalized for acute decompensation of the heart failure, and 54 were out-patients. Of the outpatient group, 25 people had implanted a left ventricular assist device (LVAD) at the time of data collection.

Our patients were suffering from advanced heart failure, predominantly with severely reduced ejection fraction (HFrEF) of various aetiologies – dilated cardiomyopathy and ischemic heart disease were present in most patients.

All patients had a comprehensive medical evaluation with clinical and laboratory assessments including echocardiography, electrocardiography and spiroergometry. In addition, the patients in the hospitalized group undergone also right-sided heart cathetrization and answered The Minnesota Living with Heart Failure Questionnaire (MLHFQ). All patients had ID parameters assessed from blood samples: TSAT, ferritin, serum iron and soluble transferrin receptor (sTfR). We also analysed correlations of ID with haemoglobin, creatinine and serum N-terminal pro-B-type natriuretic peptide (NT-proBNP).

### Biological tests and assessment of ID status and anaemia

In defining the prevalence of ID, we compared the occurrence of ID by the older definition according to the Guidelines for the diagnosis and treatment of HF from 2016, when ID in HF was defined as a ferritin value < 100 µg/l, or 100–299 µg/l if transferrin saturation (TSAT) is < 20%, with the occurrence according to the new definition.

The new definition is based on 2023 Focused Update of the 2021 ESC Guidelines, where ID in HF was defined by presence of TSAT < 20% or ferritin < 100 µg/l [17].

Anaemia was defined as haemoglobin levels < 130 g/l for men and < 120 g/l for women, as recommended by the World Health Organization [18].

### Statistical analysis

Analysis of variance (ANOVA) with Tukey post hoc test was employed to compare continuous variables in the three groups, i.e. patients with LVAD, patients without LVAD requiring hospitalization and those without LVAD recruited at our out-patient department.

Chi-square (χ^2^) test was used for categorical data, with a series of post hoc Fisher exact tests followed Bonferroni correction. The correlations between continuous variables were assessed using the Pearson correlation coefficient. Logarithmic or square root transformation was performed on continuous variables, and the normality of their distributions was checked by the Kolmogorov–Smirnov test. In the case of the questionnaire score, non-parametric Spearman correlations were used due to the multi-modal distribution. Correlations of ordinal data (such as NYHA class) was assessed using Kendall’s τ. Each analysis was then followed by Benjamini–Hochberg procedure for multiple testing correction, with false discovery rate set at 0.05. Only the results with the p-value lower than adjusted α were considered significant and are marked with * (asterisk) in the tables.

Prior to the multiple comparison adjustments, the study was powered to reveal the moderate-sized correlations (|*r*|> 0.4) with a power of 0.82 in the group of hospitalized patients, which was the largest one. In the two other groups, the power to detect the moderate-sized effects was 0.53 or 0.59, respectively, while the power to detect strong correlations (|*r*|> 0.6) exceeded 0.9 in all the three groups. In the case of ANOVA, the power to find medium-sized effects (i.e. RMSSE = 0.25) was approximately 0.5, while the study was adequately powered to reveal large effects (RMSSE = 0.40) with a power of approximately 0.8 (*α* = 0.05 is assumed in all cases). While it is difficult to estimate the power following Benjamini–Hochberg procedure, empirically, this procedure removed 27% of the results with *p* < 0.05 as non-significant.

The ability of various factors to predict severe heart failure defined as NYHA III or NYHA IV was finally assessed by a logistic regression; further, a receiving operator characteristic (ROC) curve was constructed based on sensitivity and specificity of concrete continuous predictor values, and the area under curve (AUC) was determined.

## Results

According to the older definition based on ferritin, the prevalence of ID was recorded in 56% of all patients. Focusing on parameters individually, 56% of patients had TSAT < 20% and only 33% of patients had a serum ferritin < 100 µg/l. Both criteria were met by 65%, which is a prevalence of ID in our population according to the updated definition based mostly on TSAT.

**Fig. 1:**
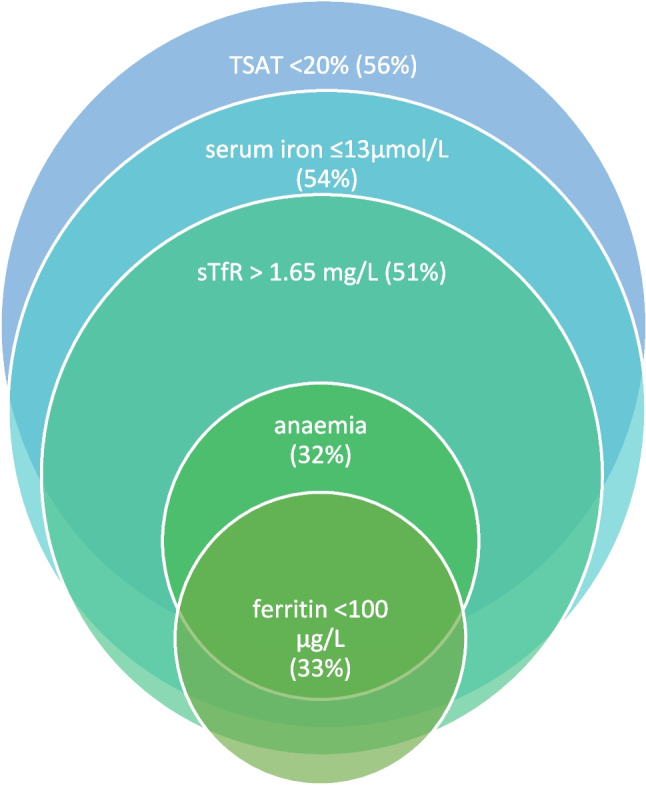
Venn diagram of the incidence of individual parameters of ID and anaemia

Parameter values in the whole group were the following (median (IQR)): TSAT was 0.18 (0.12–0.25), ferritin 128 (58.60–317.50) µg/l, sTfR 1.65 (1.27–2.10) mg/l, and serum iron was 11.9 (7.90–17.70) µmol/l. Using updated definition of ID in patients with HF, ID was present in 45% of outpatients without LVAD. In outpatients with LVAD, the prevalence of iron deficiency was 72%. The highest prevalence of ID was among patients hospitalized for decompensated heart failure, at 73% (Fig. 1).

NT-proBNP and NYHA were statistically significantly higher in hospitalized patients and in patients with LVAD compared to out-patients without LVAD (for both *p* < 0.001). In the same time, there was no significant difference in iron parameters among the groups. However, we noticed that there was a higher occurrence of anaemia in the hospitalized group compared to out-patients without LVAD (gender-specific definition of anaemia was employed) as well as lower levels of haemoglobin in patients with LVAD compared to out-patients without LVAD (*p* < 0.001). The groups further differed in terms of the levels of C-reactive protein and the levels of white blood cells, where, interestingly, the hospitalized group had the highest number of leukocytes count but the lowest CRP in the same time. We also studied the presence of diabetes in each group and found no statistically significant difference in iron parameters between patients with and without diabetes. The results are shown in Table 1.

**Table 1:** Basic characteristics of hospitalized patients, out-patients without LVAD and with LVAD. (ID was defined according to updated definition)

Variables	All patients (102)		Hospitalized (48)		Out-patients without LVAD (29)		LVAD (25)		*P*-value (ANOVA, χ^2^ test)	
ID, n ***(%)***	**66 *(65%)***		**34 *(71%)***		**13 *(45%)***		**19 *(76%)***		**0.029**	
Age at inclusion, years	**59.3 ± 11.2**		**58.7 ± 11.5**		**56.0 ± 11.5 (II)**		**64.2 ± 8.8 (I)**		0.012 *	
Gender, women, n ***(%)***	22 *(21%)*		13 *(27%)*		7 *(24%)*		2 *(8%)*		0.12	
CV characteristics										
NYHA Class III-IV vs. I–II n ***(%)***	**58 *(56%)***		**32 *(67%)* (II)**		**7 *(24%)* (I, III)**		**19 *(76%)* (II)**		< 0.001 *	
LVEF, (%)	**23.5 ± 10.6**		**24.0 ± 10.4 (III)**		**29.0 ± 11.4 (III)**		**16.0 ± 3.5 (I, II)**		< 0.001 *	
Laboratory parameters										
T-sat	0.18 (0.12–0.25)		0.16 (0.12–0.23)		0.23 (0.12–0.31)		0.19 (0.15–0.24)		0.16	
Ferritin (µg/L)	127 (51–318)		149 (72–312)		128 (59–431)		98 (46–146)		0.51	
sTfR (mg/L)	1.65 (1.27–2.09)		1.69 (1.31–2.15)		1.53 (1.16–1.87)		1.97 (1.38–2.20)		0.13	
Serum iron (μmol/L)	11.9 (8.0–17.7)		10.3 (8.2–15.8)		15.3 (8.0–19.5)		12.5 (7.9–16.0)		0.21	
CRP (mg/L)	**4.50 (1.80–12.20)**		**5.20 (1.65–20.50) (II)**		**2.40 (1.00–2.40) (I, III)**		**8.20 (4.30–13.00) (II)**		< 0.001 *	
NT-proBNP (pg/mL)	**1788 (726–5438)**		**4296 (1569–7785) (II)**		**762 (282–1361) (I, III)**		**2300 (1153–2675) (II)**		< 0.001 *	
Hemoglobin: all (g/l)	**138 ± 23**		**138 ± 23**		**149 ± 21 (III)**		**136 ± 21 (II)**		< 0.001 *	
Hemoglobin: men Anaemia: Hb < 130g/L (%)	*140* ± *23 (34%)*		*134* ± *23 (II)* *(51%)*		*152* ± *21 (I, III) (5%)*		*137* ± *21 (II) (35%)*		< 0.001	
Hemoglobin: women Anaemia: Hb < 120 g/L (%)	*130* ± *21 (23%)*		*128* ± *23 (31%)*		*136* ± *18 (14%)*		*166* ± *6 (0%)*		*0.49*	
Anaemia (%)	**32 *(31%)***		**22 *(46%) (II)***		**2 *(7%) (I)***		**8 *(32%)***		< 0.001 *	
White blood cells (10^3^/μl)	**7.0 (5.5–8.8)**		**6.8 (5.6–8.5)**		**8.1 (7.1–9.6) (III)**		**5.4 (4.2–6.7) (II)**		< 0.001 *	
Creatinine (μmol/l)	115 (91–137)		115 (95–138)		94 (77–116)		112 (98–126)		0.11	
Estimated GFR	**1.00 ± 0.40**		**0.98 ± 0.38**		**1.18 ± 0.46**		**0.76 ± 0.11**		** < 0.001**	
Diabetes presence, n ***(%)***	34 *(33%)*		14 *(29%)*		9 *(31%)*		11 *(44%)*		0.10	
Baseline medication potentially affecting iron metabolism, n ***(percentage)***										
Anticoagulation^a^		39 *(38%)*		11 *(20%)*		25 *(86%)*		3 *(12%)*		**< 0.001**
Antiaggregation^b^		19 *(17%)*		11 *(20%)*		8 *(28%)*		-		0.11
Anticoagulation + antiaggregation^c^		26 *(25%)*		3 *(6%)*		1 *(3%)*		22 *(88%)*		**< 0.001**
SGLT2i^d^		51 *(50%)*		23 *(48%)*		10 *(34%)*		13 *(52%)*		0.23

All continuous variables are presented as the median (interquartile range) unless otherwise specified.

* *P*-value significant after multiple testing correction according to Benjamini–Hochberg algorithm.

I: *p* < 0.05 vs. hospitalized group; II: *p* < 0.05 vs. out-patients without LVAD; III: *p* < 0.05 vs. patients with LVAD.

^**a**^ Various medication: warfarin, apixaban, edoxaban, rivaroxaban, dabigatran, nadroparin, enoxaparin. For hospitalized patients, we report chronic medication before admission.

^**b**^ Various medication: acetylsalicylic acid, clopidogrel, ticagrelor. Only one patient was treated with dual antiplatelet therapy at the time of data collection.

^**c**^ Warfarin + acetylsalicylic acid, rivaroxaban + clopidogrel. Reported proportion of outpatients with LVAD dominates.

^**d**^ Note: It has been found that there is a relationship between the use of sodium-glucose co-transporter-2 inhibitors (SGLT2i) and iron parameters. This relationship has not yet been fully elucidated and there are several explanations. It seems more likely that inflammation status affects iron metabolism by reducing levels of the protein hepcidin, which influences the utilization of stored iron. Thus, it is suggested that SGLT2i may reduce functional iron deficiency (19–21).

The iron-related parameters did not differ according to the primary cause of heart failure in the patients without LVAD (Table 2).

**Table 2:** Iron-related parameters according to the primary diagnosis, patients with LVAD excluded

Parameter	Hypertrophic CMP (*n* = 4)	Inflammatory CMP (*n* = 12)	IHD (*n* = 26)	Dilated CMP (*n* = 35)	*P*-value (ANOVA, χ^2^ test)
T-sat	0.11 (0.07–0.15)	0.19 (0.16–0.29)	0.19 (0.12–0.24)	0.17 (0.12–0.27)	0.12
Ferritin (µg/L)	238 (31–430)	130 (46–313)	125 (93–269)	171 (81–358)	0.91
sTfR (mg/L)	2.27 (1.33–4.81)	1.65 (1.08–1.99)	1.52 (0.20–2.01)	1.59 (1.20–2.02)	0.88
Serum iron (μmol/L)	7.9 (5.5–8.3)	13.6 (11.9–20.8)	11.2 (8.0–16.4)	11.4 (7.9–18.9)	0.068
ID, n *(%)*	4 (100%)	7 (58%)	14 (54%)	22 (63%)	0.36

Abbreviations: *CMP*, cardiomyopathy; *IHD*, ischaemic heart disease, *CRP*, C‐reactive protein; *sTfR*, soluble transferrin receptor; *NT-proBNP*, N‐terminal (NT)‐pro hormone brain natriuretic peptide; *R*, correlation coefficient; *TSAT*, transferrin saturation.

**Fig. 2:**
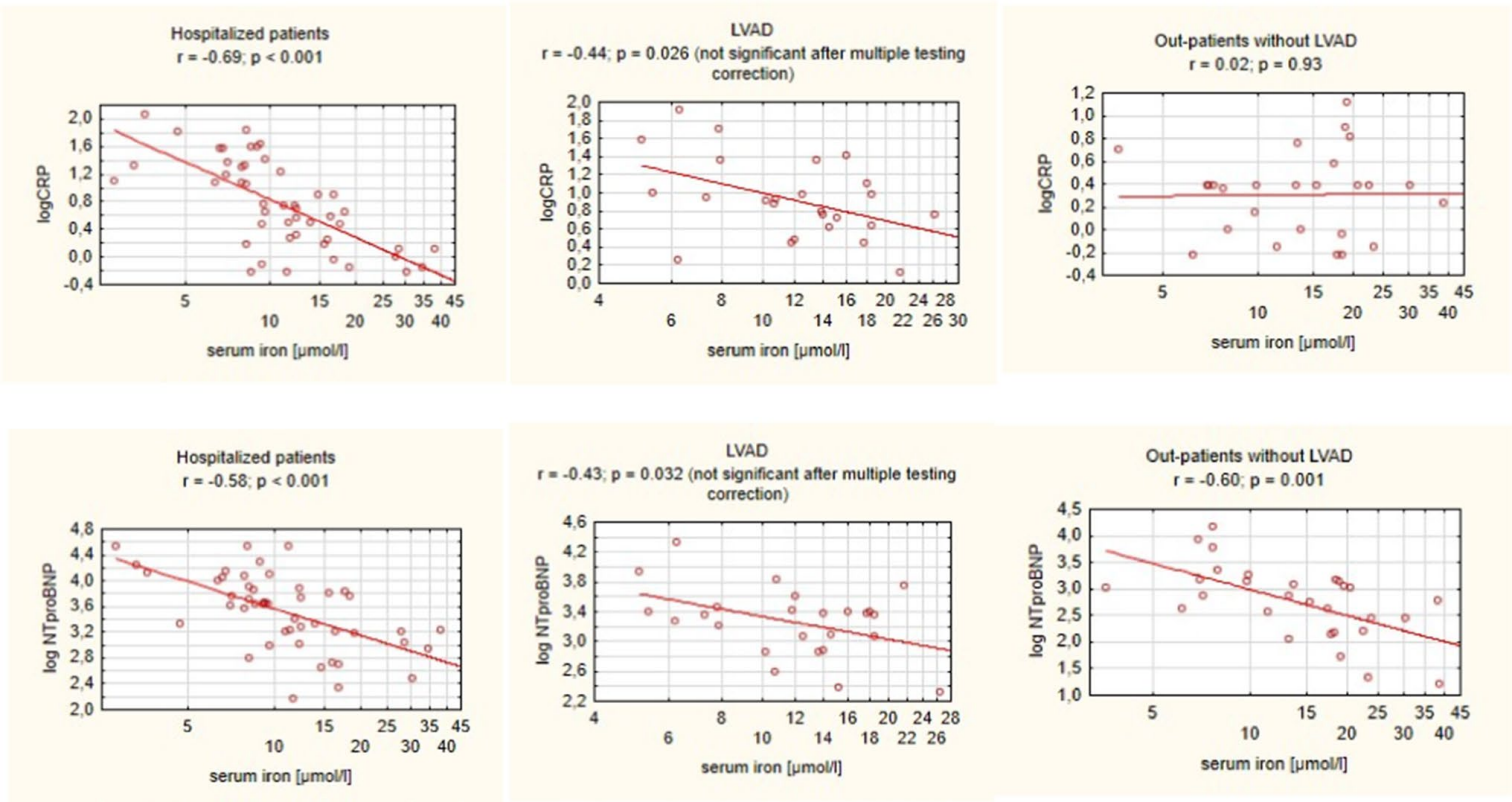
Scatter plots depicting correlations between serum iron with CRP and NT-proBNP for hospitalized patients, patients with and without LVAD

We also calculated the correlations of each ID parameter with NT-proBNP, CRP, leukocytes and estimated glomerular filtration rate (eGFR) as markers of cardiac decompensation, inflammatory state and kidney function (Fig. 2). In both groups without LVAD, we found that NT-proBNP negatively correlated with serum iron (*r* =  − 0.58, *p* < 0.001 in hospitalized patients, *r* =  − 0.60, *p* < 0.001 in out-patients), and positively correlated with sTfR (*r* = 0.47, *p* = 0.001 in hospitalized patients, *r* = 0.54, *p* < 0.002 in out-patients). The results are shown in Table 3. Furthermore, there was a negative correlation between NT-proBNP and transferrin saturation in hospitalized patients (*r* =  − 0.50, *p* < 0.001). Correlations of CRP and eGFR with ID parameters were significant only in the hospitalized group following the multiple comparison correction: CRP vs. transferrin saturation: *r* =  − 0.52, *p* < 0.001; CRP vs. serum iron: *r* =  − 0.69, *p* < 0.001.

**Table 3:** Correlations of iron metabolism parameters with cardiac, inflammatory and renal markers

Correlations (*p*-values)	Parameter	sTfR	T-sat	Serum iron	Ferritin
Hospitalized (48)	NT-proBNP	**0.47 (*p* = 0.001)**	− 0.50 (*p* < 0.001)*	− 0.58 (*p* < 0.001)*	0.10 (*p* = 0.49)
	CRP	0.36 (*p* = 0.012)	− 0.52 (*p* < 0.001)*	− 0.69 (*p* < 0.001)*	0.20 (*p* = 0.18)
	Leukocytes	0.09 (*p* = 0.54)	0.08 (*p* = 0.58)	0.11 (*p* = 0.46)	0.04 (*p* = 0.79)
	eGFR	− 0.26 (*p* = 0.076)	0.27 (*p* = 0.069)	0.41 (*p* = 0.004)*	0.08 (*p* = 0.60)
Out-patients without LVAD (29)	NT-proBNP	0.54 (*p* = 0.002)*	− 0.29 (*p* = 0.13)	− 0.60 (*p* = 0.001)*	** − 0.39 (*p* = 0.035)**
	CRP	0.11 (*p* = 0.57)	0.05 (*p* = 0.80)	0.02 (*p* = 0.93)	0.28 (*p* = 0.80)
	Leukocytes	0.13 (*p* = 0.49)	− 0.17 (*p* = 0.37)	** − **0.21 (*p* = 0.29)	0.17 (*p* = 0.37)
	eGFR	− 0.32 (*p* = 0.090)	0.09 (*p* = 0.63)	**0.39 (*p* = 0.037)**	0.12 (*p* = 0.52)
LVAD (25)	NT-proBNP	0.06 (*p* = 0.76)	− 0.33 (*p* = 0.10)	** − 0.43 (*p* = 0.032)**	0.47 (*p* = 0.019)
	CRP	− 0.12 (*p* = 0.58)	− 0.11 (*p* = 0.60)	** − 0.44 (*p* = 0.026)**	0.12 (*p* = 0.58)
	Leukocytes	− 0.24 (*p* = 0.25)	0.04 (*p* = 0.86)	** − **0.16 (*p* = 0.43)	0.19 (*p* = 0.36)
	eGFR	0.20 (*p* = 0.34)	0.15 (*p* = 0.48)	0.24 (*p* = 0.24)	** − **0.13 (*p* = 0.53)

Abbreviations: *CRP*, C-reactive protein; *sTfR*, soluble transferrin receptor; *NT-proBNP*, N-terminal (NT)-pro hormone brain natriuretic peptide; *R*, correlation coefficient; *TSAT*, transferrin saturation. Statistically significant correlations are highlighted in bold.

* *P*-value significant after multiple testing correction according to Benjamini–Hochberg algorithm.

In the subgroup of hospitalized patients, we also analysed the correlations between iron status markers and right heart catheterization parameters. In this analysis, sTfR parameter correlated most strongly with mean pulmonary artery pressure (MPAP), (*R* = 0.44; *p* = 0.003). STfR also tended to correlate in a similar manner with PCWP; however, this correlation was not significant after multiple testing correction (Table 4).

**Table 4:** Correlations of iron metabolism parameters with right heart catheterization

R (*p*-values)	**MPAP**	**PCWP**	**CVP**	**CI**
sTfR	0.44 (*p* = 0.003) *	0.39 (*p* = 0.01)	0.25 (*p* = 0.10)	− 0.26 (*p* = 0.099)
TSAT	− 0.26 (*p* = 0.093)	− 0.13 (*p* = 0.42)	− 0.13 (*p* = 0.41)	0.21 (*p* = 0.16)
Serum iron	− 0.28 (*p* = 0.071)	− 0.12 (*p* = 0.44)	− 0.18 (*p* = 0.26)	0.25 (*p* = 0.11)
Ferritin	− 0.01 (*p* = 0.95)	0.01 (*p* = 0.95)	0.19 (*p* = 0.22)	− 0.05 (*p* = 0.77)

Abbreviations: *sTfR*, soluble transferrin receptor; *TSAT*, transferrin saturation; *MPAP*, Mean Pulmonary Artery Pressure; *PCWP*, Pulmonary Capillary Wedge Pressure; *CVP*, central venous pressure; *CI*, cardiac index; *R*, Pearson correlation coefficient.

* *P*-value significant after multiple testing correction according to Benjamini–Hochberg algorithm.

Soluble transferrin receptor (sTfR) positively correlated with NYHA class (*τ* = 0.34, *p* < 0.001), while the correlation of NYHA and transferrin saturation (*τ* =  − 0.32, *p* = 0.002) and serum iron levels (*τ* =  − 0.36, *p* < 0.002) was negative. There was no significant correlation of spiroergometry outcomes (VE/VCO_2_ slope, RER, VO_2_peak ml/kg) with iron status markers (Table 5).

**Table 5:** Correlations of iron metabolism parameters with performance parameters

R/τ (*p*-values)	**NYHA class**	**VE/VCO**_**2**_** slope**	**RER**	**V O2peak ml/kg**
sTfR	0.34 (*p* < 0.001) *	0.10 (*p* = 0.57)	0.05 (*p* = 0.74)	− 0.07 (*p* = 0.69)
TSAT	− 0.32 (*p* = 0.002) *	− 0.10 (*p* = 0.59)	− 0.17 (*p* = 0.33)	0.12 (*p* = 0.47)
Serum iron	− 0.36 (*p* < 0.001) *	− 0.12 (*p* = 0.51)	− 0.10 (*p* = 0.58)	0.22 (*p* = 0.19)
Ferritin	0.01 (*p* = 0.90)	− 0.01 (*p* = 0.97)	− 0.20 (*p* = 0.26)	− 0.05 (*p* = 0.78)

Abbreviations: *sTfR*, soluble transferrin receptor; *TSAT*, transferrin saturation; *NYHA*, New York heart association; *VE/VCO2*, minute ventilation/carbon dioxide production; *RER*, respiratory exchange ratio; *VO2peak*, maximum rate of oxygen consumption; *R*, Pearson correlation coefficient; *τ*, Kendall’s tau rank correlation coefficient.

There was no significant correlation between Minnesota Living with Heart Failure questionnaire (MLHF) score and any of the iron status parameters (data not shown).

**Fig. 3:**
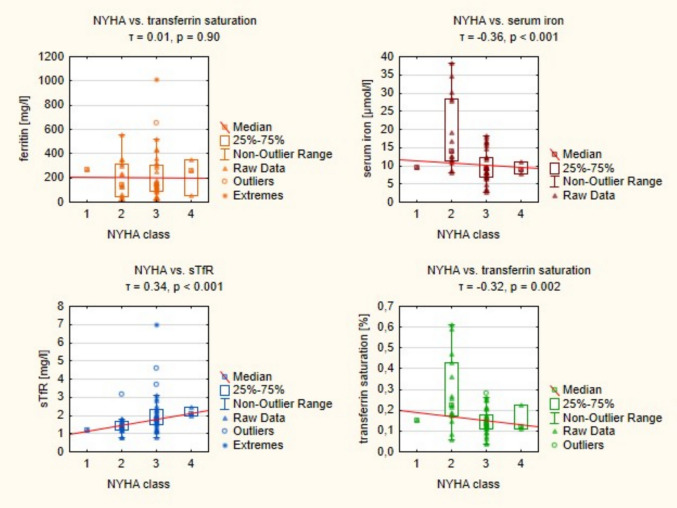
Box plots depicting correlations between iron parameters and NYHA scale for all patients

Finally, the potential of individual iron parameters to predict performance impairment in terms of NYHA III and NYHA IV (severe heart failure) was studied, and the logistic regression method was used to assess this. Receiver operator characteristic (ROC) curves were constructed based on the specificity and sensitivity of specific parameters (Fig. 3). The area under curve (AUC) was determined to define the suitability of the parameter (Table 6).

**Table 6:** Area under ROC of the individual parameters related to NYHA status

**In relation to** **NYHA**		**AUC**		
	**All (** ***n*** ** = 102)**	**Hospitalized (** ***n*** ** = 48)**	**Out-patients (** ***n*** ** = 29)**	**LVAD (** ***n*** ** = 25)**
sTfR	71.4%	76.1%	64.6%	71.1%
TSAT	70.3%	74.2%	74.0%	54.4%
Serum iron	75.7%	80.1%	72.7%	73.7%
Ferritin	59.0%	50.4%	64.9%	71.9%

Abbreviations: *AUC*, area under curve of specificity and sensitivity based on logistic regression analysis; *NYHA*, New York Heart Association; *sTfR*, soluble transferrin receptor; *TSAT*, transferrin saturation; *Youden’s point*, proposed cut-off value according to ROC curves set to NYHA-defined performance.

According to ROC curves, serum iron was the best predictor in patients with severe heart failure, while ferritin generally performed the poorest, which corresponded to its lack of correlation with NYHA class.

## Discussion

Our study supports previous experience with the high prevalence of ID in patients with HF. According to the definition of ID according to the 2023 Updated Guidelines for diagnosis and treatment of heart failure [17], 65% of patients in our study group had iron deficiency. ID was present in 45% of out-patients without LVAD, in out-patients with LVAD, the prevalence of iron deficiency was 72%. The highest prevalence of ID was among patients hospitalized for decompensated heart failure, at 73%. If we use the older definition, the prevalence of ID would be the same for out-patients without LVAD (45%), in group of hospitalized patients it would be just 63% and in the group of LVAD patients it would be 65%.

Applying updated definition, the prevalence is higher, because the diagnostic criteria have been expanded. In the previous definition, patients with ferritin > 300 μg/L were excluded (> 25% of our study patients had values above this level). Above, we can see that the differences are especially evident in the group of hospitalized and LVAD patients. In the out-patients without LVAD, ferritin levels better followed the other iron parameters. The group with low TSAT with a higher ferritin might be a group with a very good clinical response to intravenous iron supplementation, although they have been excluded from clinical trials so far [22].

Regarding haemoglobin and anaemia, ID was more frequently expressed by anaemia in hospitalized patients, especially in the male population. In hospitalized patients also enter other factors besides ID that affect the blood count, especially anaemia from blood loss. The prevalence of the presence of anaemia was higher in males in general, because although haemoglobin is significantly lower in females, there is a higher threshold for the definition of anaemia in males.

The role of medication, especially anticoagulation therapy, which was more frequent in our group of hospitalized patients, is significant and in the population of heart failure patients and participates in the worsening of iron deficiency. Interestingly, the out-patient group had a lower prevalence of iron deficiency despite the proportion of LVAD patients who had a combination of antiplatelet and anticoagulation therapy at the time of data collection. As regards medication, during recruitment of data for the study, SGLT2i entered the treatment of heart failure. Recent publications report that treatment with SGLT2i can also affect iron metabolism — it leads to changes in iron parameters that could reflect improved iron utilization. Meta-analyses explain the decrease of ferritin and TSAT and increase of sTfR in patients treated with SGLT2i as an increased iron mobilization. As the explanation is assumed to be the effect of SGLT2i on reducing the hepcidin, thereby improving the absorption and release of iron from stores for utilisation for erythropoiesis [19–21].

We found that all parameters — surprisingly except for ferritin — correlated with CRP; sTfR correlated best with the hemodynamic results obtained by right heart catheterization. Serum iron and TSAT were the most specific parameters related to functional status according to the NYHA classification.

From the results, we can deduce that the worse the level of heart failure compensation is, the worse the iron parameters are. It has long been known that heart failure is an inflammatory condition and therefore we can assume that with worse heart failure compensation, there is also worsening of functional iron deficiency. The TSAT and serum iron indicators correlate inversely with NT-proBNP and CRP, whereas sTfR correlated positively. Ferritin as the only parameter did not correlate significantly in in-patient group. This fact may generate the hypothesis that ferritin does not clearly predict iron status because of the great variability in the pathophysiological mechanisms by which it is released into the plasma [14, 16, 23].

We hope that our work could contribute to the ongoing debate about the role of ferritin in the diagnosis of iron deficiency in patients with HF and about the indication for intravenous supplementation.

Evidence from large, placebo-controlled FCM trials suggests that patients with low TSAT benefited more from FCM treatment [24]. Recently, a large meta-analysis which pooled patient data from three randomized, placebo-controlled trials of FCM in adult patients (CONFIRM-HF, AFFIRM-AHF, and HEART-FID) was presented. These three trials included a total of 4501 subjects.

In the FCM group of the HEART-FID study, the mean (± SD) TSAT at study baseline was 23.9% ± 11.2, which is considerably higher than TSAT of 15.2% ± 8.3 at baseline in the AFFIRM-AHF study and the median of 15% (IQR 11–20) at the baseline of the IRONMAN study [9–11]. Subgroup meta-analyses found that patients with the lowest transferrin saturation (TSAT < 15%) benefited more from FCM administration than those with higher baseline TSAT values [25].

These findings confirmed the importance of the debate on the reliability of ferritin as the primary marker for ID. Ferritin, unlike other markers, did not correlate with any of the right heart catheterization parameters and, in hospitalized patients, it was the only marker that did not correlate with current NYHA-defined performance status. Furthermore, from the sensitivity and specificity tests, it emerged as the least reliable parameter to define ID and performed worst overall in the in-patient group. Looking at the proposed cut-off value according to ROC curves set to NYHA-defined performance (Youden’s point), in hospitalized patients a relatively high value of 199 µg/L and below should be the most appropriate to define ID. This is probably based on the fact, that in cardiac decompensation, there is a higher inflammatory activity and ferritin is released from cells by mechanisms not related to iron storage but as an inflammatory parameter.

A study that used the gold standard of bone marrow staining to quantify an amount of iron showed that patients with HF who meet the definition with a low serum ferritin level but TSAT of more than 20% may not exhibit ID on bone marrow staining and have a lower risk of hospitalization for HF. This study suggested that TSAT ≤ 19.8% or serum iron ≤ 13µmol/L might be the most reliable markers of ID in HF [26].

A recent study investigated the association between ID and all-cause mortality in myocardial infarction (AIM) suggested that using ferritin as a marker of ID after AIM might be inappropriate, because it seems that there is an association between high ferritin and AIM, with ferritin being a product of damaged cells [23, 27]. According to a study that followed patients with ID after the implantation of resynchronization therapy (ICD-CRT), which found that iron supplementation added to ICD-CRT implantation improves LV EF and exercise capacity, it was also reported that the most significant difference in LV EF improvement was in patients who have a TSAT < 20% [28].

Regarding serum iron, according to our results, the best intersection of sensitivity and specificity was in patients with decompensated heart failure. For now, the recent evidence is not as broad, but according to a study examining the prognosis of patients after acute decompensation of heart failure, low serum iron was an independent predictor of poor prognosis regardless of ferritin and haemoglobin levels [29].

According to our results, the sTfR parameter correlated most strongly with the results of right heart catheterization and was the most reliable indicator of ID in out-patients. This supports the results of a study in which iron parameters were measured in patients with ischemic HF and bone marrow was analysed. Here, an elevated sTfR was the best predictor of the incidence of ID and also of 3-year mortality [30].

Given the new findings from recent studies and meta-analyses, there has been a gradual re-evaluation of indication criteria and changes in the evaluation of iron parameters. If intravenous iron is given to patients as indicated by ferritin, those with “too” high ferritin but low TSAT will not meet the indication criteria for supplementation. In contrast, those with low ferritin but normal TSAT may be iron given unnecessarily. According to the latest expert statements, ID in patients with HF should be defined as TSAT < 20% if ferritin is < 400 μg/l until new evidence is available, and ferritin level < 100 μg/l should not be used as a separate diagnostic criterion [16].

### Summary of clinically relevant information

The results of the study show that iron parameters are closely correlated with the degree of HF compensation. In patients with advanced HF, worse iron parameter values were associated with worse clinical status. TSAT and serum iron correlated inversely with biomarkers of decompensation (NT-proBNP, CRP), while sTfR correlated positively. In contrast, ferritin showed variability and was not a reliable indicator in the hospitalized group.

An important finding is that TSAT and serum iron are more reliable diagnostic and prognostic markers of ID than ferritin, especially in acutely decompensated patients. According to recent studies, lower TSAT (< 20%) predicts greater benefit from intravenous iron therapy and poorer prognosis in advanced heart failure, whereas ferritin may be influenced by inflammation and may not reliably reflect iron stores. The study also shows that high ferritin levels may not exclude ID, especially in patients with a pro-inflammatory state such as acute heart failure (e.g. LVAD patients).

Based on these findings, determination of ID by TSAT and serum iron appears clinically relevant for diagnosis, risk estimation, and indication of iron supplementation, whereas ferritin alone may be misleading.

### Study limitations

Firstly, during the data collection, SGLT2i, which is known for its effect on iron metabolism parameters (decreases ferritin and TSAT, increases sTfR), were added to the treatment of HF. Consequently, interpretation of differences between groups may be complicated by heterogeneity in treatment and time effect. Some changes in laboratory parameters may also be due to this therapy.

In the group of hospitalized patients, there were a very small number (units of patients, included in the variance of CRP) with an acute inflammatory state. These patients could represent a potential bias in the interpretation of iron markers in the setting of concurrent inflammation.

Adjustments to anticoagulation/antiplatelet therapy: during hospital admissions, some patients had temporary substitution of existing anticoagulation or antiplatelet therapy with low molecular weight heparin (LMWH), e.g. due to interventional procedures or instability. Due to the lack of accurate data on the frequency and duration of these modifications, it is not possible to fully evaluate their impact on the parameters studied.

Next, this is a single-centre study with a relatively small number of patients with advanced HF included mostly, limiting the generalisability of the results to a wider population.

Another limitation is, that values of a key regulator of iron metabolism, hepcidin, were not obtained, knowledge of which would allow better interpretation of the relationship between iron parameters and inflammatory activity.

Finally, we can see the differences in the subjective assessment of patients' condition by questionnaire and spiroergometry performance compared with other objective parameters — this suggests that there might be some bias in assessing correlations with patients' functional capacity.

## Conclusion

Patients with more advanced HF and worse compensation status were found to have worse ID parameters. Ferritin seems to be the ID marker with the lowest specificity, while the detection of ID using TSAT seems to be reliable. The correlation of ferritin with functional status or haemodynamics was the weakest of all the parameters examined. We suggest the revalidation of serum iron in the future, as well further investigations into the sTfR marker for the identification HF patients who might benefit from iron supplementation.

## Data Availability

The data that support the findings of this study are available from the corresponding author upon reasonable request. The data are not publicly available due to privacy restrictions.

## Abbreviations

*CRP*: C‐reactive protein
*HF*: Heart failure
*GFR*, glomerular filtration rate: *ID*, Iron deficiency
*LVAD*: Left ventricle assist device
*LVEF*: Left ventricular ejection fraction
*NT-proBNP*: N‐terminal (NT)‐pro hormone brain natriuretic peptide
*NYHA*: New York Heart Association
*sTfR*: Soluble transferrin receptor
*T-sat*: Transferrin saturation
